# A Thiopurine Drug Inhibits West Nile Virus Production in Cell Culture, but Not in Mice

**DOI:** 10.1371/journal.pone.0026697

**Published:** 2011-10-24

**Authors:** Pei-Yin Lim, Julie A. Keating, Spencer Hoover, Rob Striker, Kristen A. Bernard

**Affiliations:** 1 Department of Pathobiological Sciences, School of Veterinary Medicine, University of Wisconsin-Madison, Madison, Wisconsin, United States of America; 2 New York State Department of Health, Wadsworth Center, Albany, New York, United States of America; 3 William S. Middleton Memorial Veteran's Hospital, Madison, Wisconsin, United States of America; 4 Department of Medicine, University of Wisconsin, Madison, Wisconsin, United States of America; Kantonal Hospital St. Gallen, Switzerland

## Abstract

Many viruses within the *Flavivirus* genus cause significant disease in humans; however, effective antivirals against these viruses are not currently available. We have previously shown that a thiopurine drug, 6-methylmercaptopurine riboside (6MMPr), inhibits replication of distantly related viruses within the *Flaviviridae* family in cell culture, including bovine viral diarrhea virus and hepatitis C virus replicon. Here we further examined the potential antiviral effect of 6MMPr on several diverse flaviviruses. In cell culture, 6MMPr inhibited virus production of yellow fever virus, dengue virus-2 (DENV-2) and West Nile virus (WNV) in a dose-dependent manner, and DENV-2 was significantly more sensitive to 6MMPr treatment than WNV. We then explored the use of 6MMPr as an antiviral against WNV in an immunocompetent mouse model. Once a day treatment of mice with 0.5 mg 6MMPr was just below the toxic dose in our mouse model, and this dose was used in subsequent studies. Mice were treated with 6MMPr immediately after subcutaneous inoculation with WNV for eight consecutive days. Treatment with 6MMPr exacerbated weight loss in WNV-inoculated mice and did not significantly affect mortality. We hypothesized that 6MMPr has low bioavailability in the central nervous system (CNS) and examined the effect of pre-treatment with 6MMPr on viral loads in the periphery and CNS. Pre-treatment with 6MMPr had no significant effect on viremia or viral titers in the periphery, but resulted in significantly higher viral loads in the brain, suggesting that the effect of 6MMPr is tissue-dependent. In conclusion, despite being a potent inhibitor of flaviviruses in cell culture, 6MMPr was not effective against West Nile disease in mice; however, further studies are warranted to reduce the toxicity and/or improve the bioavailability of this potential antiviral drug.

## Introduction

The family *Flaviviridae* consists of three genera – *Flavivirus*, *Pestivirus* and *Hepacivirus*. The genus *Flavivirus* contains multiple important viral causes of human morbidity and mortality. For example, the four serotypes of dengue virus (DENV-1, -2, -3 and -4) infect more than 50 million people annually [1], and West Nile virus (WNV) can cause severe neurologic disease with an encephalitic case fatality rate of 18% [2]. Furthermore, there are no effective antivirals against any of the flaviviruses.

Drugs that alter or inhibit purine metabolism such as ribavirin and non-nucleoside inhibitors of inosine monophosphate dehydrogenase (IMPDH) inhibit flaviviruses in cell culture, but have little to no effect in animal models of flavivirus infection [3], [4], [5]. GTP specifically is required for viral translation, transcription and replication at higher concentrations than other nucleotide triphosphates [6]. The thiopurine class of modified bases and nucleosides [azathioprine, 6-mercaptopurine and 6-methylmercaptopurine riboside (6MMPr), but not thioguanine] inhibits replication of members of the *Flaviviridae* family with greater inhibition of bovine viral diarrhea virus (BVDV; genus *Pestivirus*) and hepatitis C virus (HCV; genus *Hepacivirus*) replicon than yellow fever virus (YFV; genus *Flavivirus*) replicon [7], [8]. The mechanisms by which modified nucleosides inhibit replication of RNA viruses include chain termination due to incorporation of modified nucleosides into viral RNA, direct inhibition of viral polymerase, inhibition of viral RNA capping, and mutagenesis of viral RNA [9], [10], [11], [12], [13]. Modified nucleotides that lack 3′ hydroxyls are incapable of chain extension, but at least some nucleotides with 3′ hydroxyls can be incorporated into nucleic acids and decrease the speed of extension or functionally inhibit the polymerase process in more subtle ways [14]. Clinical data suggest that the thiopurine, azathioprine, is more protective than the IMPDH inhibitor, mycophenolate, against HCV-mediated liver damage after liver transplantation [15], [16], [17]. Here we showed that viral production of both DENV and WNV was inhibited by 6MMPr although DENV was more sensitive than WNV in cell culture. Despite the sensitivity of WNV in cell culture, in a mouse model of WNV infection, 6MMPr administration did not reduce morbidity or mortality and resulted in higher viral loads in the brains of mice.

## Results

### 6MMPr inhibits flavivirus production in multiple cell lines

**Figure 1:**
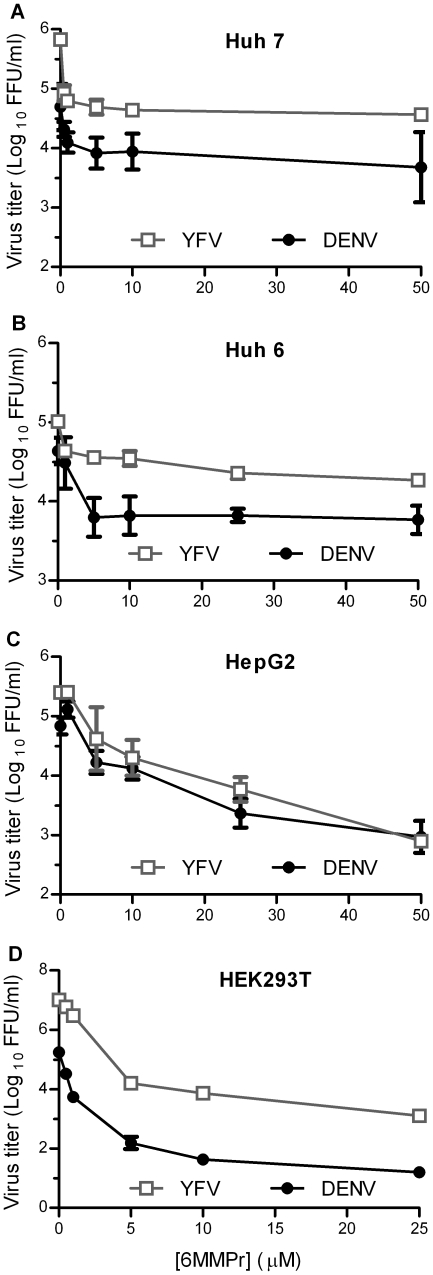
6MMPr inhibited DENV and YFV production in multiple cell lines in a dose-dependent manner. (A) Huh7, (B) Huh6, (C) HepG2, and (D) HEK293T cells were inoculated with DENV-2 (16681) or YFV in the presence of various concentrations of 6MMPr. Medium was harvested at 48 hpi, and viral titers were determined by fluorescent focus assays. At least two independent experiments were performed, and the means ± standard deviations from one experiment, performed in triplicate, are shown.

We previously showed that 6MMPr is a potent inhibitor of HCV replicon and BVDV in Huh7 cells [8]. We extended these results by examining the effect of 6MMPr on viral growth for members of the *Flavivirus* genus (DENV, YFV and WNV) in several cell lines. Human hepatic and kidney cell lines were inoculated with DENV-2 or YFV in the presence of various concentrations of 6MMPr, and virus production was measured at 48 hours post-inoculation (hpi). Similar to our previous results for BVDV, 6MMPr inhibited viral production for DENV-2 and YFV by approximately 10-fold in Huh7 cells (Figure 1A). In addition, 6MMPr reduced viral production by 10-fold in Huh6 cells (Figure 1B), 100-fold in HepG2 cells (Figure 1C), and 10,000-fold in HEK293T cells (Figure 1D). The greater inhibition of viral production in HEK293T cells was not due to drug cytotoxicity in these cells (data not shown), which is consistent with our previous results demonstrating that 6MMPr up to 500 µM does not cause cytotoxicity in Madin-Darby bovine kidney cells [8].

**Figure 2:**
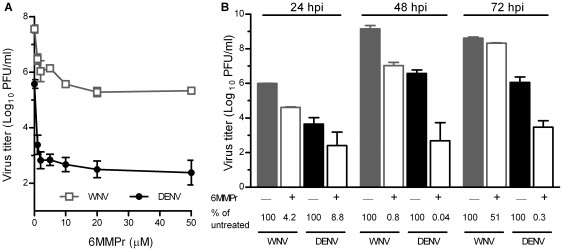
6MMPr inhibited DENV production to a greater extent than WNV production. Vero cells were inoculated with WNV or DENV-2 (New Guinea C) in the presence of 6MMPr at (A) various concentrations or at (B) 10 µM. Medium was harvested at (A) 48 hpi or (B) various times pi, and viral titers were determined by plaque assays. At least two independent experiments were performed, and the means ± standard deviations from one experiment, performed in (A) sextuplets or (B) triplicates, are shown. The antiviral effects on WNV and DENV-2 were compared at each concentration of 6MMPr in (A), and *P* values were ≤0.005 at all concentrations tested (1–50 µM).

We compared the antiviral effect of 6MMPr against DENV-2 and WNV – two distantly related flaviviruses. 6MMPr inhibited viral production for both DENV-2 and WNV in a dose-dependent manner at 48 hpi in Vero cells (Figure 2A). At maximum inhibition (20–50 µM 6MMPr), DENV-2 was inhibited 1000-fold, and WNV was inhibited 100-fold. At all concentrations tested, 6MMPr inhibited viral production for DENV-2 to a greater extent than for WNV by approximately 10-fold (p<0.005), suggesting that 6MMPr is a more effective inhibitor of DENV than WNV. We confirmed these results by treating cells with 10 µM 6MMPr and measuring production of infectious DENV-2 and WNV at various times post-inoculation (pi). 6MMPr had a similar effect on DENV-2 and WNV at 24 hpi, but inhibited DENV production 10- to 100-fold more than WNV production at 48 and 72 hpi, respectively (Figure 2B). In conclusion, 6MMPr inhibited viral production of two distantly related flaviviruses, and the efficiency of inhibition was virus and cell type dependent. DENV-2 was more sensitive to 6MMPr than WNV, and for DENV production, 6MMPr caused 10- to 100-fold greater inhibition in the two kidney cell lines (HEK293T and Vero) than in the two liver cell lines (Huh 7 and Huh 6), which may reflect differences in cell growth or inherent differences in cell types.

### Treatment of mice with 6MMPr

We explored the use of 6MMPr as an antiviral against WNV in a well-characterized immunocompetent mouse model, using the highly susceptible C3H/HeN (C3H) mouse strain [18]. A previous report showed that BUB mice tolerate treatment with 80 mg/kg/day 6MMPr for 10 consecutive days [19]; therefore, we opted to test a lower dose to avoid toxicity. We treated C3H mice once per day with vehicle alone or 0.5 mg 6MMPr (approximately 25 mg/kg/day) intraperitoneally (IP) for 10 days, and no clinical signs of toxicity or weight loss greater than 5% were observed (data not shown). 6MMPr has a short half-life in mice [20]; therefore, we tested twice per day treatment in a subsequent study to attempt to maintain drug levels. Mice were treated twice per day with vehicle alone, 0.25 mg 6MMPr (approximately 25 mg/kg/day; low dose group), or 0.5 mg 6MMPr (approximately 50 mg/kg/day; high dose group) for seven consecutive days, and mice were weighed daily. Weight loss of greater than 10% was observed in all of the mice in the high dose group and half of the mice in the low dose group, but no weight loss was observed in the vehicle treated group (data not shown). These results suggest that once per day treatment with 0.5 mg 6MMPr is just below a toxic dose for C3H mice, and this dose was then used in subsequent antiviral studies.

**Figure 3:**
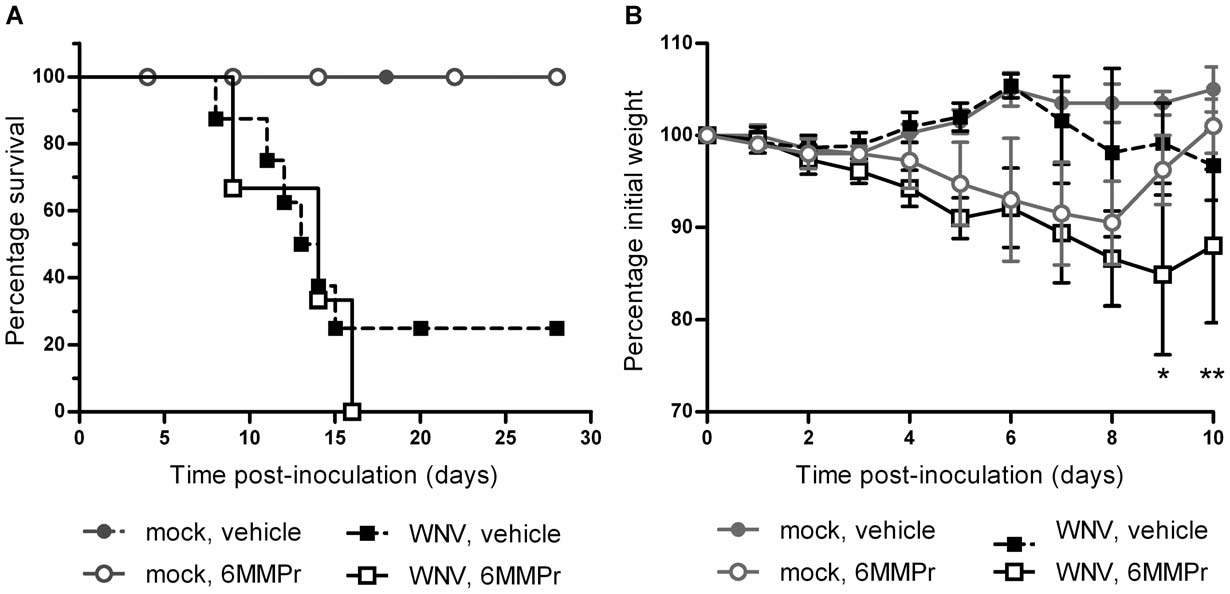
6MMPr treatment of mice did not improve survival and exacerbated weight loss during WNV infection. Adult, female C3H mice were treated with vehicle or 0.5 mg 6MMPr once daily for eight consecutive days (days 0 to 7 pi). Immediately after the first treatment, mice were inoculated SC in the left rear footpad with diluent alone (mock; n = 4 per group) or with 10^3^ PFU of WNV (n = 8 per group) and monitored daily for weight loss and clinical signs. (A) Survival data are shown. Viral infection was confirmed in all WNV-inoculated survivors by seroconversion to WNV. (B) The means ± standard deviations of percentage initial weight are shown. Significant *P* values between mock-inoculated, 6MMPr-treated mice and WNV-inoculated, 6MMPr-treated mice are indicated as follows: *, *P*<0.05; **, *P*<0.005.

We determined the effect of 6MMPr on morbidity and mortality following subcutaneous (SC) inoculation with 10^3^ PFU of WNV with treatment starting immediately prior to virus inoculation. Mice were treated once daily with vehicle control or 0.5 mg 6MMPr for eight consecutive days (days 0–7 pi relative to WNV inoculation). All WNV-inoculated mice (vehicle-treated and 6MMPr-treated) exhibited clinical signs of disease, and there were no significant differences in disease onset or mortality between the vehicle-treated and 6MMPr-treated mice (Table 1). Furthermore, treatment with 6MMPr had no significant effect on survival of the WNV-inoculated mice (*P* = 0.75) (Figure 3A). 6MMPr treatment resulted in weight loss in both the mock-inoculated and WNV-inoculated mice with an average 10% weight loss after eight daily treatments, and 6MMPr treatment exacerbated weight loss due to West Nile disease, starting on day 8 pi (Figure 3B). These results demonstrate that treatment with 6MMPr did not reduce morbidity or mortality due to West Nile disease and resulted in mild toxicity manifested as weight loss.

**Table 1:** Treatment with 6MMPr did not affect morbidity or mortality in WNV-inoculated mice.

Virus inoculum	Treatment	Clinical Signs (no. sick/total)	Average disease onset (SD) (days)	Mortality (no. died/total)	Average survival time (SD) (days)
Diluent	Vehicle	0/4	NA	0/4	NA
Diluent	6MMPr	0/4	NA	0/4	NA
WNV	Vehicle	8/8	9.2 (1.3)	6/8^1^	11.8 (2.5)^2^
WNV	6MMPr	8/8	9.9 (2.2)	8/8	12.1 (2.4)

Adult, female C3H mice were inoculated SC in the left rear footpad with diluent or 10^3^ PFU WNV. Mice were treated for eight days with vehicle or 0.5 mg 6MMPr IP once per day starting immediately prior to virus inoculation. Mice were monitored for weight loss and clinical signs.

1 Two mice that survived WNV infection seroconverted to WNV by day 28 pi.

2 Only mice that died were included in the calculation. NA = not applicable. SD = standard deviation.

**Figure 4:**
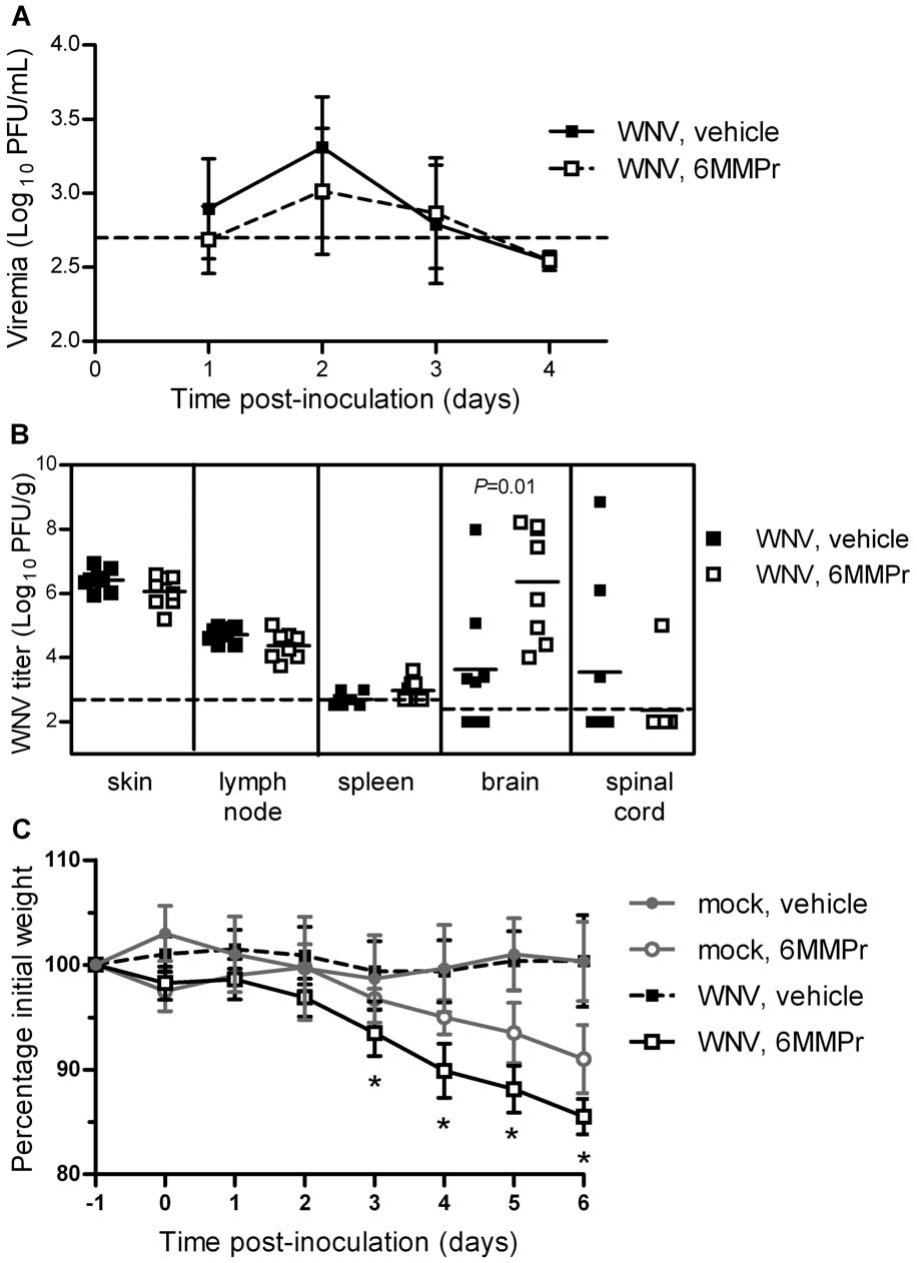
The effect of 6MMPr treatment of mice during WNV infection was tissue dependent. Adult, female C3H mice were pre-treated with vehicle or 6MMPr once daily for seven consecutive days (days −1 to 5 pi). On day 0, mice were inoculated SC with diluent alone (mock; n = 3–4 per group) or 10^3^ PFU of WNV (n = 8 per group). Mice were weighed daily, serially bled three times between days 1–4 pi (for WNV-inoculated mice, n = 4 per group on days 1 and 4 pi and n = 8 per group on days 2 and 3 pi), and euthanized on day 6 pi when tissues were harvested. (A) The geometric means ± standard deviations of virus titers in sera are shown. (B) Viral loads in tissues on day 6 pi are shown. Each data point represents an individual mouse, and the horizontal solid lines represent the geometric means. (C) The means ± standard deviations of the percentage initial weight are shown, and significant *P* values between mock-inoculated, 6MMPr-treated mice and WNV-inoculated, 6MMPr-treated mice are indicated: *, *P*<0.05. For (A) and (B), significant *P* values between vehicle and 6MMPr treated mice inoculated with WNV are noted on the graphs, and the dashed lines represent limits of detection of (A) 500 PFU/mL or (B) 500 PFU/g for skin, draining lymph nodes and spleen and 250 PFU/g for brain and spinal cord. Data points below the dashed lines are less than the limit of detection for the plaque assay.

Possible explanations for the lack of effectiveness against West Nile disease by 6MMPr include low drug concentrations during early WNV infection and/or poor bioavailability in the central nervous system (CNS). Thus, we conducted a study to examine the effect of 6MMPr pretreatment on viral loads in the periphery and CNS. Mice were pretreated with vehicle or 0.5 mg 6MMPr once per day for seven consecutive days, starting one day prior to viral inoculation, and viral titers were determined in serial bleeds from days 1, 2, 3, and 4 pi and in tissues harvested on day 6 pi. The 6MMPr-treated group had a 2-fold lower geometric mean titer at peak viremia (2 days pi) than the vehicle-treated group, but the difference was not significant (*P* = 0.085) (Figure 4A). Treatment with 6MMPr resulted in significantly higher viral loads in brains (*P* = 0.013), but not in spinal cords, skin at the inoculation site, draining lymph nodes, and spleen (Figure 4B). Weight loss was again observed in the 6MMPr-treated mice with significantly greater losses on days 3–6 pi in the WNV-inoculated mice compared to the mock-inoculated mice (*P*<0.05) (Figure 4C), which was most likely due to the combined stress of viral infection, serial bleeding, and drug treatment. In conclusion, treatment with 6MMPr had no significant effect on viremia or viral loads in the periphery, but it enhanced viral loads in the brain, suggesting that the effect of 6MMPr is tissue dependent in the mouse model.

## Discussion

In this work we have extended our observations that not only pestiviruses (e.g. BVDV) and hepaciviruses (e.g. HCV) are inhibited by thiopurines in mammalian cell culture [7], [8], but several distantly related flaviviruses (e.g. DENV-2, YFV and WNV) are as well. We previously presented data showing that the HCV replicon is more susceptible to inhibition by 6MMPr than a YFV replicon, and our current studies with the complete life cycle of YFV showed that YFV had similar susceptibility as DENV-2 (Figure 1). We tested multiple DENV-2 strains, including 16681 (Figure 1), New Guinea C (Figure 2), and S1 (data not shown), and a DENV-4 strain (data not shown), and there was no difference in sensitivity between DENV strains. On the other hand, DENV-2 was 10-fold more sensitive than WNV to 6MMPr treatment. One potential explanation for this difference is that the replication kinetics of WNV are faster than DENV and, therefore, less altered by the effect of 6MMPr on cell growth. Another possibility that is not mutually exclusive with the effect on cell growth is that there are small differences between the conserved DENV and WNV polymerase GTP binding/priming pocket [21], [22], postulated to regulate replication. Numerous groups have shown that non-nucleoside IMPDH inhibitors, such as mycophenolic acid, inhibit the flaviviruses DENV, YFV, and WNV [23], [24], [25], but susceptibility differences between flaviviruses have not been reported. While both mycophenolic acid and thiopurines have cytostatic effects and block purine synthesis, their mechanisms differ. Thiopurines decrease purine synthesis through inhibition of glutamine-5-phosphoribosylpyrophosphate aminotransferase [26], and mycophenolic acid inhibits purine synthesis by inhibiting IMPDH [27].

Although DENV is more susceptible to thiopurine inhibition in cell culture, we chose to test the effect of 6MMPr on WNV in a mouse model because infection of mice with WNV is a well-characterized, relatively inexpensive immunocompetent system from which both mortality and viral load data can be obtained. Treatment with 6MMPr had no significant effect on production of infectious virus in the skin at the inoculation site, draining lymph node, spleen and spinal cord, but resulted in significantly greater viral titers in the brain. Possible explanations for these tissue differences include low drug concentrations in the brain, poor efficacy of 6MMPr in neurons of the brain, and disruption of the blood brain barrier by 6MMPr, changing the kinetics of neuroinvasion. Future experiments are required to explore these possibilities.

We were surprised both by the toxicity that we observed from 6MMPr in WNV-infected animals, as well as the milder toxicity/weight loss seen in the uninfected animals. 6MMPr has been administered in humans in combinations with other antimetabolite chemotherapy [28], [29], [30] as well as in mice [19] with less toxicity than we observed. The mouse strain we used (C3H) differed from the limited published literature on 6MMPr in mice [19], but other thiopurines, such as azathioprine, have been extensively used in mice without causing weight loss. Recent data show azathiaprine triggers erythropoiesis and works against malaria in cell culture and in mice [31]. 6MMPr is a downstream metabolite of azathioprine and presumably would have similar toxicity, but perhaps 6MMPr is less toxic when produced *in situ* from a precursor. After liver transplants, whether patients have HCV or not, they are given a cocktail of two to three immunosuppressants to prevent rejection that typically includes either the IMPDH inhibitor, mycophenolate, or the thiopurine, azathioprine. It is unclear whether the differences between these two types of immunosuppressants on HCV posttransplant [14], [15], [16] are due to their effect on cellular biosynthetic pathways or differences in the degree to which the immune system is suppressed.

In conclusion, despite the potent antiviral effects of 6MMPr on flaviviruses in cell culture, in a mouse model of WNV, 6MMPr exacerbates weight loss and increases viral loads in the brain, possibly due to poor bioavailability in the central nervous system. While 6MMPr may be useful in probing differences in replication among flaviviruses, clearly more selective antiviral activity is necessary for safe therapy for WNV. Finally, future development as an antiviral against DENV is warranted since DENV is rarely neurotropic, and 6MMPr was more efficacious in cell culture against DENV, suggesting a greater therapeutic window.

## Materials and Methods

### Ethics statement

Mouse studies were performed under strict accordance of the criteria established by the National Institutes of Health, and all efforts were made to minimize suffering. The protocols were approved by the Institutional Animal Care and Use Committees of the Wadsworth Center (protocol #09-377) or the University of Wisconsin-Madison (protocol #V01452).

### Cells

African green monkey kidney cells (Vero; ATCC #CCL-81) and baby hamster kidney cells (BHK-21; ATCC #CCL-10) were maintained in minimum essential medium (Gibco-Invitrogen, Carlsbad, CA) supplemented with 10% heat-inactivated fetal bovine serum (FBS) , 2 mM L-glutamine, 1.5 g/L sodium bicarbonate, 100 U/ml penicillin, and 100 µg/ml streptomycin. Human embryonic kidney cells (HEK 293T; ATCC #CRL-11268), human hepatocellular carcinoma cells HepG2 (ATCC #59194) and Huh7 (JCRB0403), and human hepatoblastoma cells Huh6 (JCRB0401) were maintained in Dulbecco's modified Eagle medium (Gibco-Invitrogen) supplemented with 10% FBS, 100 U/ml penicillin, and 100 µg/ml streptomycin. *Aedes albopictus* mosquito cells (C6/36; ATCC #CRL-1660) were maintained in L-15 medium (Gibco-Invitrogen) supplemented with 10% FBS, 100 U/ml penicillin, and 100 µg/ml streptomycin. All mammalian cells were incubated at 37°C, 5% CO_2_, and mosquito cells were incubated at 28°C, 5% CO_2_.

### Viruses

WNV was produced from a full-length cDNA clone of a 2000 New York strain by electroporation of BHK-21 cells with *in vitro* transcribed RNA as previously described [32]. Viral titers of WNV were determined by plaque assay on Vero cells. DENV-2 strain New Guinea C and DENV-2 strain 16681 (Genbank accession number U87411) were obtained from the Centers for Disease Control and Prevention (CDC, Fort Collins, CO). DENV-2 (New Guinea C) was prepared in Vero cells as previously described [33]. DENV-2 strain 16681 was prepared by infecting C6/36 cells at a multiplicity of infection of 10 in L-15 medium with 100 U/ml penicillin and 100 µg/ml streptomycin. After 1 h incubation at 28°C, FBS was added to L-15 medium to make 2% final concentration. Supernatants were harvested on day 5 pi, clarified by centrifugation (500×g for 5 min), and stored in aliquots at −80°C. YFV strain PFLYF17D (Genbank accession number X03700) was prepared from a full-length cDNA clone [34] by transfection of Huh7 with *in vitro* transcribed RNA using Mirus TransIT RNA Transfection Kit (Mirus Bio, Madison, WI), as per manufacturer's instruction. Viral titers of DENV-2 and YFV stocks were determined by fluorescent focus assays on Vero cells.

### Fluorescent focus assay

Fluorescent focus assay was modified from a previous report [33]. Briefly, 48-well plates were seeded with Vero cells at a density of 5×10^4^ cells/well and incubated at 37°C. After 24 hpi, cells were inoculated with 10-fold serial dilutions of virus for 1 h at 37°C, and then an overlay medium containing 0.5% agarose, 0.5× Earle's Balanced Salt solution, 0.033% Yeast Extract, 0.165% lactalbumin hydrolysate, 4% FBS, 0.225% sodium bicarbonate, 50 U/ml penicillin, and 50 µg/ml streptomycin was added. After 48 h at 37°C, cells were fixed with 1∶1 methanol∶acetone solution for 10 min and incubated with mouse ascites fluid against DENV (1∶500 dilution; Harlan, Madison, WI) or antibody against YFV (Clone 2D12.A; 1∶800 dilution; Millipore, Billerica, MA) in phosphate buffered saline (PBS) with 0.2% bovine serum albumin for 1 h at room temperature, followed by donkey anti-mouse IgG conjugated with Alexa-Fluor 488 (1/200 dilution; Invitrogen, Carlsbad, CA) for 30 min at room temperature. Fluorescent foci were observed using a Nikon Eclipse TE300 microscope (Melville, NY), and foci within the entire well were counted and expressed as focus forming units (FFU) per ml.

### Effect of 6MMPr on virus growth in cell culture

Approximately 4×10^4^ cells/well were added to 24-well plates and incubated for 24 h at 37°C. Cells were rinsed with PBS and inoculated with 10^4^ PFU or FFU of virus (multiplicity of infection of approximately 0.25 based on titration on Vero cells). Immediately after the addition of virus, 6MMPr (Sigma-Aldrich, St Louis, MO) was added to the cultures in the appropriate medium without FBS. After 1 h incubation at 37°C, FBS was added to make a 2% final concentration of FBS, and cells were incubated at 37°C. At various times pi, supernatants were collected and stored at −80°C. Virus production was determined by fluorescent focus assays or plaque assays on Vero cells.

### Antiviral studies in mice

Five-week-old, female C3H mice were purchased from Taconic (Germantown, NY), acclimatized for at least 1 week in the biosafety-level-3 animal facility, and given food and water ad libitum. 6MMPr was dissolved in sterile PBS (tissue culture grade; Invitrogen) immediately before treatment of mice for all studies. Mice were inoculated IP with 100 µl of sterile PBS (vehicle control) or 100 µl of 6MMPr once or twice per day at the designated dose. Mice were weighed and clinically assessed daily. Percentage initial weight was calculated to evaluate weight loss, and 10% weight loss was considered significant. No clinical signs of drug toxicity, such as ruffled fur, hunching, weakness, labored breathing, ascites or abdominal irritation, were observed in any of the studies.

For the morbidity and mortality study, six- to seven-week-old C3H mice (14.7–19.7 g, mean weight of 17.4 g) were treated with PBS (vehicle control) or 6MMPr (0.5 mg/mouse) once daily for eight consecutive days. Immediately after the first treatment, mice were inoculated SC in the left rear footpad with diluent alone (mock; n = 4 per group) or 10^3^ PFU of WNV (n = 8 per group) as previously described [18]. Mice were monitored daily for weight loss and clinical signs, and mice with severe disease were euthanized. Clinical signs included ruffled fur, hunching, weakness and ataxia. A mouse was considered to have clinical West Nile disease if at least one of the following criteria was met: 1)≥10% weight loss; 2) clinical signs for at least two days. WNV infection was confirmed in all WNV-inoculated survivors by the detection of antibodies to WNV as previously described [18].

For evaluation of viral loads in sera and tissues, six- to seven-week-old C3H mice (16.6–19.8 g, mean weight of 18.3 g) were treated with PBS or 6MMPr (0.5 mg/mouse) for seven consecutive days, and treatment was begun one day prior to viral inoculation. Mice were inoculated with diluent (n = 3–4 per group) or WNV (n = 8 per group) as described above, weighed daily, and serially bled three times via puncture of the maxillary vein. Half of the mice in each group was bled on days 1, 2 and 3 pi, and the other half was bled on days 2, 3 and 4 pi, resulting in 4 mice per group on days 1 and 4 and 8 mice per group on days 2 and 3. On day 6 pi, mice were euthanized, and tissues were harvested and stored at −70°C. Tissues were processed as previously described [18], and viral loads in sera and tissues were determined by plaque assays on Vero cells. Infectious virus was not detected in mock-inoculated mice.

### Statistical analyses

A two-tailed Mann-Whitney *U* test (GraphPad, San Diego, CA) was used to test for differences between the effect of 6MMPr on DENV and WNV growth in cell culture, viral loads in serum and tissues, and weight loss. Values of 333 PFU/ml for serum and peripheral tissues and 100 PFU/g for brain and spinal cord were used to calculate the geometric means and standard deviations for any samples below the limit of detection for the plaque assays. A log-rank survival analysis (GraphPad) was used to test for the effect of treatment on survival. For all analyses, a *P* value≤0.05 was considered significant.
